# Integrative Proteomic Analysis of Serum and Peritoneal Fluids Helps Identify Proteins that Are Up-Regulated in Serum of Women with Ovarian Cancer

**DOI:** 10.1371/journal.pone.0011137

**Published:** 2010-06-15

**Authors:** Lynn M. Amon, Wendy Law, Matthew P. Fitzgibbon, Jennifer A. Gross, Kathy O'Briant, Amelia Peterson, Charles Drescher, Daniel B. Martin, Martin McIntosh

**Affiliations:** 1 Division of Public Health Sciences, Fred Hutchinson Cancer Research Center, Seattle, Washington, United States of America; 2 Institute for Systems Biology, Seattle, Washington, United States of America; Cincinnati Children's Research Foundation, United States of America

## Abstract

**Background:**

We used intensive modern proteomics approaches to identify predictive proteins in ovary cancer. We identify up-regulated proteins in both serum and peritoneal fluid. To evaluate the overall performance of the approach we track the behavior of 20 validated markers across these experiments.

**Methodology:**

Mass spectrometry based quantitative proteomics following extensive protein fractionation was used to compare serum of women with serous ovarian cancer to healthy women and women with benign ovarian tumors. Quantitation was achieved by isotopically labeling cysteine amino acids. Label-free mass spectrometry was used to compare peritoneal fluid taken from women with serous ovarian cancer and those with benign tumors. All data were integrated and annotated based on whether the proteins have been previously validated using antibody-based assays.

**Findings:**

We selected 54 quantified serum proteins and 358 peritoneal fluid proteins whose case-control differences exceeded a predefined threshold. Seventeen proteins were quantified in both materials and 14 are extracellular. Of 19 validated markers that were identified all were found in cancer peritoneal fluid and a subset of 7 were quantified in serum, with one of these proteins, IGFBP1, newly validated here.

**Conclusion:**

Proteome profiling applied to symptomatic ovarian cancer cases identifies a large number of up-regulated serum proteins, many of which are or have been confirmed by immunoassays. The number of currently known validated markers is highest in peritoneal fluid, but they make up a higher percentage of the proteins observed in both serum and peritoneal fluid, suggesting that the 10 additional markers in this group may be high quality candidates.

## Introduction

Ovarian cancer (OC) is a leading cause of suffering and death for women in the United States, and diagnosing it at a pre-metastatic stage may dramatically reduce mortality. Although OC accounts for only 4% of all cancer diagnoses in women (National Cancer Institute. http://www.cancer.gov) it is the most lethal of all gynecologic cancers. As with many cancers, a woman's survival [1] with OC is strongly associated with its stage at diagnosis. Serous ovarian cancer (SOC) is the most prevalent and deadly histology; over 70% of all OC cases are diagnosed in a metastatic stage.

Early detection strategies for OC currently under evaluation have typically involved combining one or more blood-based markers (typically the marker CA 125) as a means to refer women to a confirmatory imaging modality such as transvaginal sonography. When using a marker as a first-line screen, the performance of the entire screening strategy will be limited by the performance of this marker and a critically important performance attribute for an early detection marker is lead-time, i.e. how early in the disease process the marker elevates. Although preliminary results suggest that achieving a positive predictive value threshold of 10% [2] is feasible using the sequential multi-modal approach, modeling approaches [3]–[6] and pre-clinical validation studies profiling CA 125 and other markers [7] suggest that the lead-time obtained from CA 125 may be insufficient to meaningfully reduce mortality in a large fraction of women.

Many markers other than CA 125 have been identified and validated in independent studies using samples collected at the time of clinical diagnosis [8]–[18]. We refer to these markers as ‘validated predictive proteins’, by which we mean proteins confirmed using immunoassays in multiple independent samples and, therefore, as a group, are likely to be predominantly true positives. More recently, many of these markers have been evaluated in samples obtained prior to diagnosis and suggest that we can expect few proteins validated in symptomatic disease to also elevate before symptoms develop [7]. Clearly, improving early detection for SOC will require identification of new classes of markers, possibly by plasma or serum proteomic approaches.

One goal of our study includes identifying additional markers using serum proteomics. However, the feasibility of discovering differential proteins in serum and plasma has been controversial and not widely successful, and so a secondary goal of our study is to validate the overall serum proteome experimental workflow using several markers as gold standards. In this manuscript we describe a set of proteomic experiments that interrogate complex mixtures of human OC biomaterials. The experiments had two purposes; the first was to identify previously unidentified proteins that may be additional candidates as predictive markers. The second was to validate the serum proteomics approach by tracking the behavior of known validated predictive proteins in order to establish that the platform is capable of discovering markers. Early plasma and serum proteome discovery efforts, most often relying on SELDI or MALDI methods [19]–[26], have largely failed in this regard. More recent approaches using tandem MS combined with intensive fractionation [27]–[29] might be more appropriate for serum and plasma biomarker discovery as they have been shown to identify proteins for pancreatic cancer [30] that have been subsequently validated. However, because the success of any discovery approach will certainly depend on the disease characteristics as much as the technology, prior to investing resources into developing antibodies for new markers, we first wished to confirm that the approach is capable of producing reproducible results.

We have interrogated both a circulating fluid, serum, and a fluid proximal to the tumor, ascitic fluid or peritoneal fluid from control patients with benign serous tumors (BST). We conducted two different experiments on serum: the first experiment compared serum pools from metastatic SOC patients to pools from matched healthy asymptomatic volunteers; the second experiment compared serum pools from a different set of metastatic SOC patients to serum pools from women with BST. For the proximal fluid experiments, we compared pools of ascitic fluid from metastatic SOC patients to pools of peritoneal fluid from patients with BST. In all experiments, samples were matched based on age, storage duration, hormone replacement therapy use and time of collection prior to surgery (for case/control comparison). By combining these data sets and identifying proteins that appear to be up-regulated in SOC in both serum and fluid proximal to the tumor, we hope to enrich our potential candidate list with cancer-specific markers.

## Results

### Annotation of proteins based on existing data resources

A list of 21 proteins previously shown to be up-regulated in plasma or serum of SOC compared to healthy control by ELISA assays was compiled by examining the OC biomarker literature. We identified these proteins as well as one additional protein, IGFBP1, discovered and validated in this work as true positive controls. Four of the true positive proteins were not identified in either plasma or peritoneal fluid: MUC16 [31]–[33], TNFRSF6B [14], VTCN1 [14], [17] (aliases CA 125, DcR3, B7-H4, respectively) and VEGF [16]. Table 1 lists the 19 true positive proteins that were identified in plasma or peritoneal fluid [14], [16], [17], [31]–[33]. All of them were observed and quantified in peritoneal fluid, and 7 were observed in plasma; no positive controls were identified exclusively in plasma.

**Table 1:** Summary of performance of true positive “benchmark serum proteins.

gene symbol	alias	ref^a^	log2 (SOC/HA)^b^	log2 (SOC/BTS)^c^	SOC pert. fluid^d^	BST pert. fluid^e^	mean serum ratio^f^	pert. fluid ratio^g^
CHI3L1		[33]			69	5		13.8
ENPP2	LPA, ATX	[8]			108	21		5.1
IGFBP2		[10]	1.42	0.60	133	36	2.02	3.7
KLK10		[11]			6	0		6.0
KLK11	hk11	[12]			13	3		4.3
KLK5		[11]			2	0		2.0
KLK6		[11]			41	1		41.0
KLK7		[11]			3	0		3.0
KLK8		[11]			8	0		8.0
LCN2		[33], [47]	1.11	0.06	57	6	1.50	9.5
MIF		[33], [48]			56	117		0.5
MMP7		[33]	1.42		10	0	2.68	20.0
MSLN		[32], [33]			45	33		1.4
SLPI		[13]	0.23	1.20	5	0	1.64	10.0
SPON2		[14]			9	0		9.0
SPP1	OPN	[33], [49]			24	2		12.0
TIMP1		[15]	0.59	0.80	73	24	1.62	3.0
WFDC2	He4	[18]	1.27	2.67	22	3	3.93	7.3
IGFBP1			1.89	2.41	11	0	4.45	22.0

Proteins that have been previously shown to be up-regulated in serum or serum of SOC cases compared to HA controls by ELISA assays, or (for IGFBP1) shown for the first time here.

a) literature citing assay results.

b)log2 transformed IPAS ratio SOC/HA.

c)log2 transformed IPAS ratio SOC/BST.

d)peptide counts from SOC peritoneal ascitic fluid.

e) peptide counts from BST peritoneal fluid.

f)geometric mean of ratio measured in two IPAS experiments.

g) ratio of SOC peritoneal fluid counts to BST peritoneal fluid counts.

### Serum comparisons

**Figure 1:**
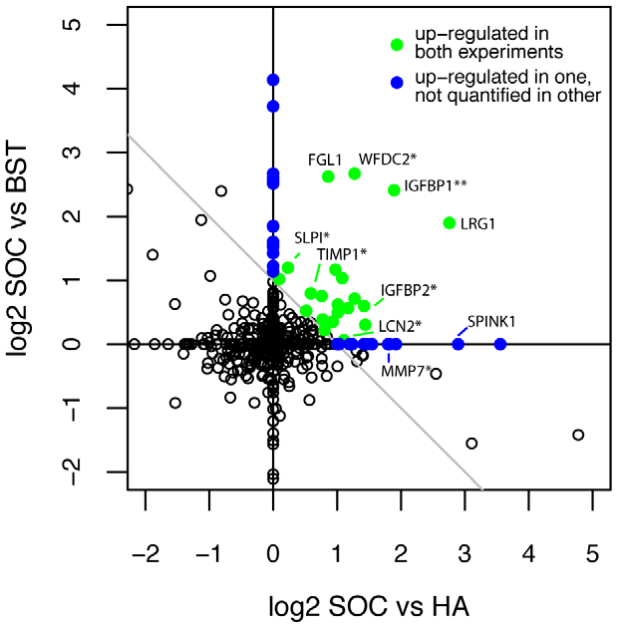
Log2 serum protein ratios from both IPAS experiments. Green points represent proteins up-regulated in both experiments. Blue points represent proteins up-regulated in one experiment and not observed in the other. *Benchmark marker validated by ELISA assay; **New marker validated by ELISA assay.

The serum proteomics experiments (described below) compared metastatic serous ovarian cancer (SOC) to either healthy asymptomatic (HA) women or women with benign serous tumors (BST). Table 2 shows the total number of quantified proteins in each serum experiment. Over 360 proteins were quantified in each experiment and a total of 470 proteins were quantified in at least one or the other experiment. Collapsing these proteins by gene symbol results in slightly lower totals due to different isoforms identified for the same gene. In Figure 1, the association between log2 ratios for all quantified proteins by gene symbol for the two serum experiments is plotted (horizontal and vertical axis reflect SOC versus HA and SOC versus BST, respectively). Note that all ratios are oriented so that a positive value implies a protein ratio higher in SOC. Proteins that were not observed in one experiment are represented by a pseudo ratio of ‘1’ (log2 = 0) for that experiment.

**Table 2:** Summary of proteins quantified in each experiment.

Experiment	Quantified Protein Groups	Quantified Gene Symbols
**Serum**		
SOC v HA	362	331
SOC v BST	374	332
Combined	470	416
**Peritoneal fluid**		
SOC	1788	1609
BST	2810	2603
Combined	3239	2950

Serum experiments count only those proteins for which a cysteine containing peptide was observed. Peritoneal fluid experiments count all proteins observed.

Figure 1 reveals a promising pattern that one can expect to see in an experiment that compares two complex mixtures and in which some differential proteins are observed: most proteins fall near the origin and vary in an uncorrelated manner since no systematic changes are occurring but a number of them in the upper right quadrant trend away from the bulk. For the purpose of characterizing our results, we label a protein as up-regulated if its consensus ratio meets or exceeds a 2-fold change (i.e, log2 (SOC/HA)+log2(SOC/BST)≥1 as indicated by the line in Figure 1). All 54 proteins meeting this criterion are shown in green if observed in both comparisons or blue if observed in only one. These proteins are also listed in Table S1 (in supplementary information).

Those proteins that correspond to our ‘benchmark’ proteins from Table 1 are denoted with an asterisk. A total of seven benchmark proteins were observed in serum including six that were previously validated as well as one additional marker, IGFBP1, denoted with a double asterisk, which we validated here based on these experiments. All seven of the observed benchmark proteins were up-regulated by our criteria. The observed enrichment (7 of 7) is highly significant (p-value = 1e-6), demonstrating that our serum proteomic analysis finds high concordance with validated assays.

### Peritoneal fluid experiments

**Figure 2:**
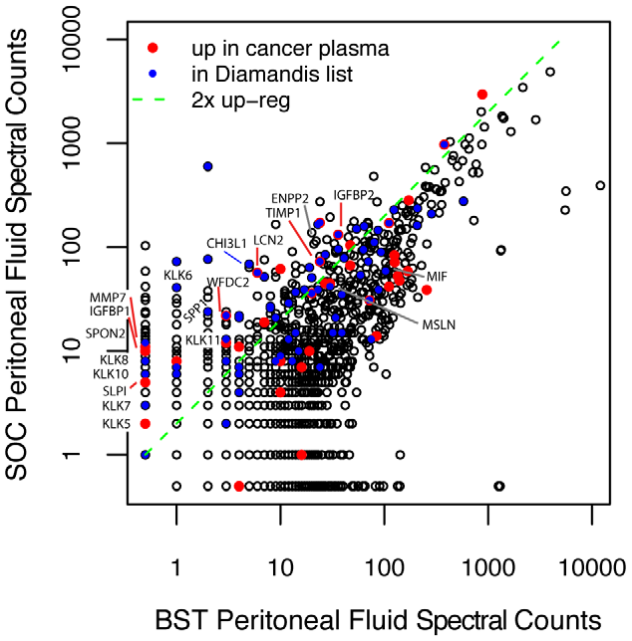
Comparison of peptide counts from peritoneal fluid experiments. Red points are up-regulated in SOC serum. Blue points are proteins observed in ovarian cancer ascitic fluid by Kuk et al [34]. Benchmark proteins are labeled. Points above green dashed line are considered up-regulated in peritoneal fluid.

The peritoneal fluid experiments are compared using a label-free method that allows us to quantify all observed proteins, not only those containing an isotopically labeled cysteine amino acid. After collapsing by gene symbol, 2950 proteins are observed in both experiments. The comparison of log2(peptide spectral counts) between SOC to BST is plotted in Figure 2. For convenience, proteins with spectral count = 0 in one fluid but are observed in the other fluid are set to 0.5. We define a protein as up-regulated if it has an SOC peptide count at least two-fold higher than the corresponding BST peptide count. From the 2950 identified proteins by gene symbol, 358 are selected as potentially up-regulated based on this criterion (see Table S1). Proteins that were also found up-regulated in the serum experiments are shown in red. We also annotate those proteins reported by Kuk *et al* [34] who evaluated SOC ascitic fluid. In our experiments, 73 of the Kuk candidates are observed, shown in blue in Figure 2, and are distributed widely among SOC and BST counts with only 37 up-regulated. Kuk *et al* did not evaluate material from BST. As in the serum experiments, we found a significant enrichment of validated biomarkers among the up-regulated proteins in ascitic fluid. Of the 19 benchmark proteins observed in either peritoneal fluid (labeled in Figure 2), all but two, MIF and MSLN, were up-regulated (p-value = 2e-16). Note that MIF up-regulation is possibly associated with sample ascertainment bias [9]. The data here are consistent with the conclusions of Kuk *et al.* that cancer ascitic fluid may be a valuable resource for identifying biomarker candidates, although our results from the BST suggest that many of them will not be cancer specific.

### Comparison of serum versus peritoneal fluid

**Figure 3:**
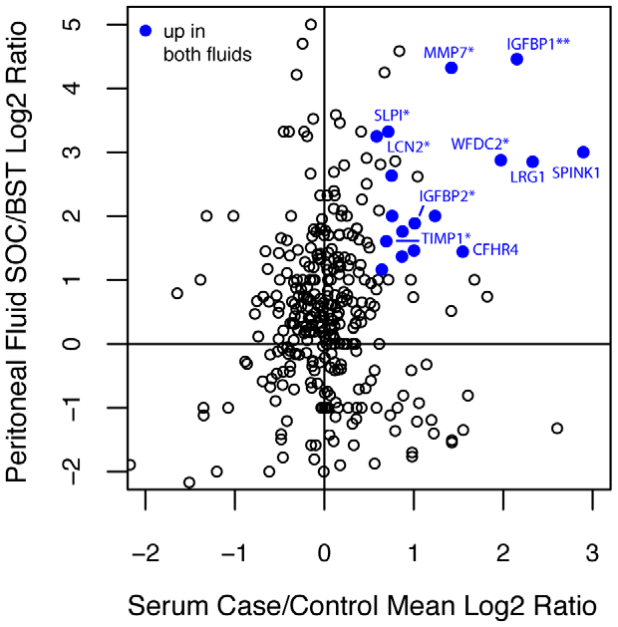
Comparison by log2 ratio of serum to peritoneal ascites fluid. Proteins up-regulated in both fluids are colored blue. *Benchmark marker validated by ELISA assay; **New marker validated by ELISA assay.

To compare the two different types of fluid, the consensus log2 ratios over both serum experiments were plotted against the log2 ratio of SOC ascitic fluid over BST peritoneal fluid, as shown in Figure 3. Because only proteins observed in both materials can be plotted, this comparison includes only those 358 proteins observed in both serum and peritoneal fluid. A total of 17 of these proteins were designated as up-regulated in both sets. The overall concordance of all proteins, as measured by the correlation in the ratios, is not strong, but this is not unexpected since most proteins are not changing between the two sources and for those experimental sources of variation should dominate. Only among proteins that systematically vary between case and control should we find concordance. The 17 proteins that are up-regulated in both experiments (listed in Table 3 and shown in blue in Figure 3) are highly enriched for the validated biomarkers listed in Table 1. All seven observed benchmark proteins measured in both experiments were found up-regulated (p-value = 2e-16). However, the greatest benefit we observe by using the overlap of the two experiments is a substantial increase the percentage of validated markers among all up-regulated markers. In serum, 15% of the up-regulated proteins were benchmark proteins and in the ascitic fluid candidate list, 5% were benchmark proteins. If we use the overlap of those two experiments, the percentage of candidate proteins from the validated benchmark list increases to 7 out of 17 (41%).

**Table 3:** Proteins up-regulated in both serum and peritoneal fluid.

genes	log2 SOC/HA	log2 SOC/BST	SOC pert. fluid spectral count	BST perit. fluid spectral count	mean serum ratio	pert. fluid SOC/BST ratio	GO extracellular	GO inflammatory/response to wounding	assay availability	validated by ELISA
CFHR4	1.55		19	7	2.92	2.71	yes			
CFHR5	0.93	0.35	105	47	1.56	2.23	yes			
GOLM1	1.01	0.49	62	10	1.69	6.20				
HAVCR2	0.76	0.75	12	3	1.69	4.00				
IGFBP1	1.89	2.41	11	0	4.45	22.00	yes		yes	yes
IGFBP2	1.42	0.60	133	36	2.02	3.69	yes		yes	yes
LCN2	1.11	0.06	57	6	1.50	9.50	yes		yes	yes
LRG1	2.76	1.90	173	24	5.03	7.21	yes			
MMP7	1.42		10	0	2.68	20.00	yes		yes	yes
MMP9	1.00		11	4	2.00	2.75	yes		yes	no
ORM1	1.44	0.30	2970	879	1.83	3.38	yes	yes		
SERPINA3	1.17	0.56	974	378	1.83	2.58	yes	yes		
SLPI	0.23	1.20	5	0	1.64	10.00	yes		yes	yes
SPINK1	2.90		8	1	7.44	8.00	yes			
TIMD4	1.24		2	0	2.36	4.00				
TIMP1	0.59	0.80	73	24	1.62	3.04	yes		yes	yes
WFDC2	1.27	2.67	22	3	3.93	7.33	yes		yes	yes

In addition to experimental data, Table 3 also includes annotation for two GO categories, extracellular region and inflammatory or response to wounding where a “yes” indicates the protein is annotated for that term either at the leaf or a parent node. The “extracellular region” term indicates that protein is located outside the plasma membrane and could be secreted into blood. Among the 17 proteins, 14 (82%) of them are annotated as extracellular compared to 17% of all proteins observed in the experiments. This disproportion supports the hypothesis that by combining data from circulating and proximal fluids we might be more likely to discover protein biomarkers secreted or shed from the tumor rather than deriving elsewhere in the host. We have also included GO terms related to inflammation in this table to rule out proteins that may not be cancer-specific. Only two of the 17 overlap proteins are known to be involved in inflammation.

### ELISA validation of IGFBP1

The validation status of all 17 candidate proteins is indicated in Table 3. Commercial ELISA assays are available for 8 of the 17 proteins. Of those, six were validated in previous studies (see Table 1). The remaining two proteins derived from this study, MMP9 and IGFBP1, were assayed in this study.

**Figure 4:**
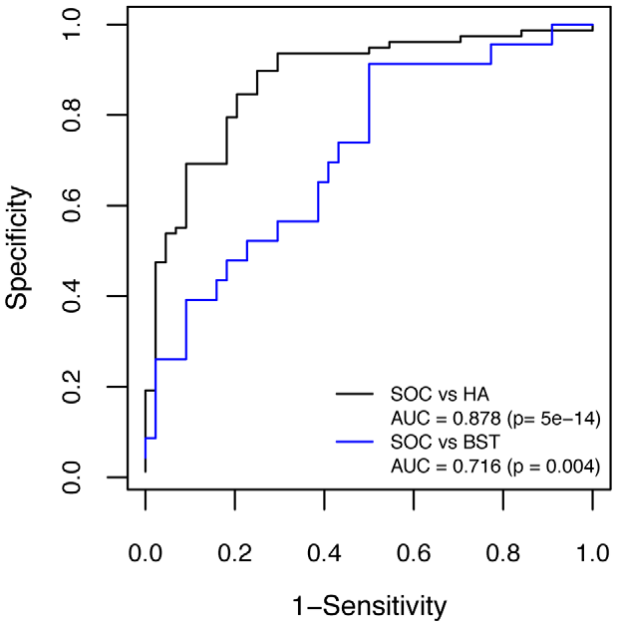
IGFBP1 ROC curves. SOC vs HA is shown in black and SOC vs BST in blue.

Validation was performed in three stages. In the first stage, the markers were tested in a series of mixtures of varying ratios of pooled sera from OC patients and pooled sera from HA controls from the filtering set of samples (50 OC, 9 HA controls) described in Methods. Both proteins had high concentrations in the high OC pool but only IGFBP1 showed a concentration dependence with the ratio of ovarian cancer sera to control. Both proteins were then tested against individual samples from the filtering set (12 OC, 12 HA controls) described in Methods. IGFBP1 levels were significantly higher in cancer serum (p-value = 0.0097) while MMP9 levels were not (p-value = 0.20). A third set of samples that was restricted to serous ovarian cancer cases (44 SOC, 78 HA controls) was used to confirm the elevation of IGFBP1. In this data set, the significance of case to control difference increased to 5.0e-4 and the ROC curve had an AUC of 0.860. The ROC curve for these samples shown in Figure 4 demonstrates the ability of IGFBP1 to classify OC versus HA and BST controls, showing results consistent with the behavior in the proteomics experiments.

## Discussion

Proteomic profiling of serum or plasma to discover cancer biomarkers has not yet met with widely demonstrated success [19]–[26] where the proteins were validated in independent samples or using independent platforms. One notable exception is a pancreatic cancer study using a mouse model and in-depth fractionation of intact proteins [30]. In this manuscript we have used a similar approach for tandem MS proteomic profiling of serum in combination with profiling of peritoneal fluid as proximal tumor fluid. We have produced a short list of 17 biomarkers found up regulated in both serum and proximal fluid – that include 7 proteins that have been validated using existing ELISA assays, including a new SOC biomarker (IGFBP1). The remaining 10 markers in this group may represent high quality candidates.

Our success in identifying many validated markers may have resulted from a number of features in the experimental design. Sample bias was eliminated by careful sample collection and tightly matching cases to controls. In addition to matching based on age, race and family history of OC, controls were also matched on HRT use and number of days prior to surgery at blood draw. HRT has been shown to dramatically affect the levels of many serum proteins [35], [36]. Thorpe *et al* [37] demonstrated that blood drawn the day of surgery is associated with the elevation of several serum proteins. This bias was avoided here by careful sample matching based on case/control collection condition. Another important aspect this analysis was the use of extensive sample fractionation prior to MS. This design, which has been shown to obtain sensitivities of 10ng/ml concentration [35], [36], allowed greater depth of proteome coverage by tandem MS to identify potentially meaningful changes.

An important aspect of the design of the serum experiments was the use of three different types of subjects: ovarian cancer patients, healthy asymptomatic volunteers and patients with benign tumors. Proteins up-regulated in SOC relative to HA but not relative to BST are not likely to be cancer-specific and were eliminated from consideration. The same was true of proteins up-regulated relative to BST but not to HA. Using only the SOC vs BST comparison, we would have found 5 out of 7 quantified validated markers as opposed to 7 out of 7 (TIMP1 and SLPI would be removed).

Combining the serum data with data from proteomic profiling of peritoneal ascitic fluid allowed us to identify a group of proteins have a large fraction of our true positive proteins; eliminating proteins not found also in peritoneal fluid reduced our potential candidates from 54 to 17 while retaining all seven benchmark proteins quantified in serum. This result supports the hypothesis that proteins identified in a fluid proximal to the tumor and also in a circulating fluid will identify effective biomarkers. Kuk *et al* [34] showed that ascitic fluid from patients with ovarian cancer contained a large number of high quality markers, and our results are concordant with those findings. However, because the number of benchmark proteins found in peritoneal fluid is higher than found in serum, one cannot make the claim that more markers can be identified by requiring observations in both serum and proximal fluid. Indeed our data and that from Kuk *et al.* suggest that ascitic fluid proteomics data may contain the greater number of high quality markers. Our findings suggest only that requiring proteins to be observed in both samples could lead to a greater proportion of high quality markers. This hypothesis cannot be confirmed, however, without also systematically evaluating true negative markers as well as true positives. Moreover, not identifying all known markers does not indicate that the marker is not of high quality nor that the process is necessarily at fault. For example, our platform was not able to identify CA 125 nor three other markers that we initially selected as benchmark proteins, possibly due to sensitivity limitations on mass spectrometry. However, given that a platform is able to quantify existing validated markers, one would find concordance between the two platforms reassuring, which we found here.

In their combined data from four different fractionation methods Kruk *et al.* identified 445 proteins total, a number considerably lower than the thousands of protein groups we observed in our experiments. This difference is partly due to their focus on the subproteome of proteins less than 100 kDa as our set includes 490 proteins larger than 100kDa. But the use of intensive fractionation may account for the remaining disparity. Despite these differences, the conclusions of their study are highly consistent with our findings; after applying some data mining techniques, they reduced their candidate list to 77 proteins (including 25 known OC markers), 73 of which were observed in our study. As apparent in Figure 2, the relative abundances of the Kuk proteins include some that are up as well as down; only half of these are up by two-fold relative to the benign peritoneal fluid. Of the 19 benchmark proteins observed in either pool of peritoneal fluid, 17 have spectral counts two-fold higher in SOC than BST. This suggests that although Kuk *et al* were correct in their assertion that ascitic fluid is a rich source of potential markers, our work suggests that the inclusion of a control material can be helpful in reducing false positives. One of the strengths of this study is the use of relative abundances of SOC to BST to find proteins specific to malignant disease.

The issue of confounding inflammatory proteins (recently addressed by Checlinska *et al* [38]) is minimized in multiple ways in our study. First, our depletion and fractionation methods allow us access to proteins beyond just the highly abundant ones that often include many proteins related to inflammation. Additionally, our comparisons between cancer and benign disease filter out many proteins that elevate due the presence of a benign tumor; inflammatory proteins shared by both conditions are eliminated. Also, since we are targeting secreted proteins by looking for overlap between proteins in the proximal fluid and those elevated in serum, we will reduce the possibility of observing serum proteins synthesized in the liver as an inflammatory response. Still, Table 3 includes two proteins (ORM1, SERPINA3) out of 17 biomarker candidates that have roles in inflammation. While we have greatly improved the proportion of biomarkers related to inflammation over previous profiling experiments [39], [40], we can not completely remove all confounding factors and must rely on annotation when available for furthering filtering.

In this work we have also discovered and validated a novel biomarker for symptomatic serous ovarian cancer, insulin growth factor binding protein 1. Like IGFBP2, IGFBP1 is a member of the insulin-like growth factor binding protein family and binds both insulin-like growth factors, IGF1 and IGF2. Like the other IGFBPs, IGFBP1 is expressed in local tissues, including ovary, and is present in normal plasma. Serum levels of IGFBP1 are significantly decreased in post-menopausal women taking hormone replacement therapy [35]. Though elevated serum levels of IGFBP2 have been demonstrated in a number of cancers, including ovarian [10], this is the first evidence that IGFBP1 levels increase in serum or plasma of patients with ovarian cancer. One other study showed elevated levels of IGFBP1 (and IGFBP2) in plasma of patients with head and neck cancers [41].

Finally, we also note that among the 17 markers shown to be up-regulated in the serum and ascitic fluid of SOC, all but three are secreted proteins. Although it is routinely claimed that secreted proteins should be preferred as candidates due to their potential to secrete into the blood, this claim is not often supported empirically. Our data provide a strong support for this generally accepted hypothesis.

As described in the introduction of this manuscript, though several serum biomarkers for ovarian cancer have been discovered and validated, all of the markers have shown poor performance in pre-diagnostic samples. In this study, we set out to establish the capability of proteomic profiling of serum to discover ovarian cancer biomarkers when used in conjunction with other profiling of proximal fluid. Having demonstrated the success of this approach, we can now confidently apply these methods to more challenging pre-symptomatic samples.

## Materials and Methods

### Recruitment and collection of human blood and peritoneal fluid for discovery

All research for this study was specifically conducted under Fred Hutchinson Cancer Research Center Institutional Review Board approved protocols, IR# 6045 and IR# 6094. All human samples were derived from subjects who provided written consent. Data were analyzed anonymously.

Serum from women with serous ovarian cancer (SOC), benign serous tumors (BST), and from healthy asymptomatic (HA) controls was used in our proteomic discovery and validation experiments. Sample collection protocols have been previously described in detail elsewhere [32], [37]. In brief, serum and peritoneal fluid from women with SOC and BST were collected prior to surgery and chemotherapy as part of an Ovarian Cancer Specialized Program of Research Excellence (SPORE) funded by the National Cancer Institute. Diagnosis of SOC and BST were confirmed by central pathology review. Age and hormone replacement therapy (HRT) matched HA controls were recruited through an ovarian cancer screening program, where all patients represent asymptomatic controls. All serum samples, regardless of the collection source, were processed with the same protocol; blood was collected in 10cc Vacutainer Serum Separator Tubes (SST) and allowed to sit for 30 minutes to 4 hours at room temperature. The tubes were centrifuged at 1200-×g for 10 minutes, then split into multiple 1-ml aliquots of serum and stored at −80 degrees Celsius. Prior to use, each 1-ml vial of serum was thawed on ice, split into multiple 110-microliter subaliquots, and stored back at −80 degrees Celsius.

Peritoneal fluid was collected into a sterile vacuum-sealed container in the operating room by surgical staff. Using a sterile 1cc syringe, 20,000 units of heparin (vials contain 10,000 units per cc) was added to each liter of fluid to prevent the formation of blood clots. In the lab, the fluid was transferred to conical centrifuge tubes (50cc or 250cc depending on fluid volume) and spun in a balanced centrifuge at 2000 rpm for 5 minutes to pellet the cellular component. Cell-free supernatant was transferred to one 50cc conical tube and five 1.5 ml microcentrifuge tubes for storage at −80 freezer degrees Celsius.

### Selection of specimens for discovery experiments

Only post-menopausal women having average risk of ovarian or breast cancer were included (average risk implies no significant family history of breast or ovarian cancer and no previous cancer diagnosis) in the discovery experiments. The first serum discovery experiment compared women with late stage SOC to HA controls and the second compared to women with BST. SOC pools of size 4 (to healthy controls) and size 5 (to BST) were selected to be tightly matched to controls based on age (+/−2 years), sample storage time (+/−1 year) and HRT [35]. Moreover, because collection environment has a known effect on serum proteome [37], cases and controls were also matched based on whether the blood collection occurred the day of surgery (for SOC versus BST controls) or whether they were collected three or more days prior to surgery (for SOC versus HA controls). For the peritoneal fluid experiments, a pool of size 10 SOC samples was compared to a pool of size 4 BST samples. The pool size for BST is smaller because, while a large fraction of SOC patients develop ascites, few BST patients produce comparable ascitic fluid.

### Selection of serum samples for validation experiments

Experiments to validate the proteins were performed for two markers in three previously described [32], [33] sets of serum specimens. These sets included two filtering sets. The first set was composed of pooled samples from 50 OC patients (case pool) serially diluted with serum from 9 HA controls (control pool). The second set was comprised of individual samples from 12 OC patients and 12 HA controls. A third larger set of individual samples was used to confirm proteins that showed significant elevation in the second set. This set was restricted to serous ovarian cancer cases and included 44 SOC patients and 78 HA controls, representing a subset of those sampled described by Palmer et al [33].

### Sample processing of serum for discovery experiments

In each case/control comparison serum was depleted of the seven most abundant proteins and quantitatively compared using isotopically labeled acrylamide [29] following extensive off-line separation of intact proteins. This method is referred to as the Intact Protein Analysis System (IPAS) protocol [28]. In brief, case and control pools were separately depleted of abundant human proteins using two Multiple Affinity Removal System (MARS)-7 high capacity columns (Agilent, Santa Clara, CA) coupled serially on a Shimadzu HPLC system following the manufacturer's protocol. Depleted samples were concentrated using Amicon-15 and Amicon-4 concentrators (Millipore, Billerica, MA) down to 100–250 ul then diluted to 0.5–0.8 ml in labeling buffer (8 M urea, 100 mM Tris pH 8.5, 0.5% octyl-beta-d-glucopyranoside (w/v)). A standard Bradford assay was performed to determine protein concentration. The samples were reduced by addition of 0.66 mg of dithiotreitol per 1 mg of protein, then incubated at room temperature for 2 hours. Samples were alklyated with acrylamide (light label) (Sigma-Aldrich, St. Louis, MO) or C13-acrylamide (heavy label) (Cambridge Isotope Laboratories, Andover, MA) by addition of 7.1 mg/mg protein of acrylamide or 7.4 mg/mg protein of C13-acrylamide per 1 mg of protein, then incubated in the dark at room temperature for 1 hour. Samples labeled with acrylamide were mixed with samples labeled with C13-acrylamide, and immediately filtered with a low protein-binding filter. In the SOC/HA serum comparison, the case pool was labeled with heavy acrylamide and control pool light acrylamide. In the SOC/BST serum comparison, the label orientation was reverse so that the case pool (SOC) had the light label. The change in label orientation was used to avoid any label bias and to serve as a means to help filter out bad identifications by selecting peptides that are observed in both heavy and light forms.

The combined mixture was subjected to an automated online 2D-HPLC system described by Piterri et al [42]. Briefly, using the Workstation Class-VP 7.4 (Shimadzu Corporation), the labeled serum was separated in the first dimension on an anion exchange column (Poros HQ/10, 10 mm i.d.×100 mm l, Applied Biosystems) using an 8 step-elution (from 0 to 1000 mM NaCl) at 0.8 mL/min. Fractions from each of the 8 anion-exchange separation elution steps were automatically transferred onto a reversed-phase column (PorosR2/10, 4.6 mm i.d.×100 mm l, Applied Biosystems) for second dimension of separation in to 84 fractions for every anion-exchange fraction. A 25 min gradient elution (from 5% to 95% mobile phase B) was used at 2.4 mL/min. Mobile phase A for anion-exchange chromatography consisted of 20 mM Tris (Sigma), 6% isopropanol (Fisher), and 4 M Urea, pH 8.5, and mobile phase B was the same composition and pH as mobile phase A with 1 M NaCl (Fisher) added. Mobile phase A for reversed-phase chromatography consisted of 95% water, 5% acetonitrile, and 0.1% TFA (Supelco), and mobile phase B consisted of 90% acetonitrile, 10% water, and 0.1% TFA.

The resulting 672 aliquots were lyophilized and resuspended in 0.25 M urea (Fisher) containing 50 mM ammonium bicarbonate and 4% acetonitrile and then digested overnight with 200 ng of modified trypsin (Promega). The digestion was interrupted by addition of 5 µL of 10% formic acid solution. Digestion was carried out overnight at 37°C. The resulting peptide mixtures were acidified with 5 µL of 1% formic acid. Each set of 84 reverse phase fractions was pooled to 12, resulting in 96 fractions total.

### Processing peritoneal fluid

Case and control peritoneal fluid were compared using a label-free approach rather than by isotopic labeling. Each case and control pool was separately depleted of the seven highest abundant serum proteins using two MARS-7 columns as above. As with serum, two dimensions of fractionation were used. First intact proteins were separated into 12 fractions by reversed phase then each fraction was digested prior to a second orthogonal separation using isoelectric focusing. Specifically, digested reversed-phase separated fractions of cancer and benign peritoneal fluid were each combined into six pools of approximately 330 µg peptide per pool. Each pool was desalted on a C_18_ column (Waters, Milford, MA), eluted in 80% acetonitrile in acidified water, and dried under reduced pressure. Each pool was individually prepared for separation by isoelectric focusing using the Agilent 3100 OFF-GEL Fractionator per the manufacturer's protocol. Briefly, each dried peptide sample was resuspended in a solution containing OFF-GEL sample buffer and pH 3–10 ampholytes (Agilent), and divided into 24 wells over a rehydrated immobilized pH 3–10 gradient gel (Agilent). With cancer and benign samples on independent sample trays, pools were simultaneously focused for 33 hours until the accumulation of ∼56 kVh. Focused fractions were then combined in groups of two to form 12 fractions for each of the 12 original pools. The digested sample was desalted on a Grace (Deerfield, IL) Vydac UltraMicroSpin C18 cartridge and dried prior to mass spectrometric analysis.

### Mass spectrometry acquisition

Each fraction was analyzed for each experiment by a LTQ-Orbitrap (Thermo) mass spectrometer coupled with a NanoLC-1D (Eksigent). The liquid chromatography separation was performed in a 25 cm column (Picofrit 75 µm i.d., New Objectives, packed in-house with MagicC18 resin) using a 90 min linear gradient from 5 to 40% of acetonitrile in 0.1% formic acid at 300 nL/min for shotgun analysis. Spectra were acquired in a data-dependent mode in *m*/*z* range of 400–1800, including selection of the 5 most abundant +2 or +3 ions of each MS spectrum for MS/MS analysis. Mass spectrometer parameters were capillary voltage of 2.0 kV, capillary temperature of 200°C, resolution of 60 000, and target value of 1 000 000.

### Mass spectrometry interrogation and data processing to Identify and quantify proteins and to aligning results from experiments

The acquired LC-MS/MS data were searched against the human International Protein Index (IPI) version 3.48 using the Mascot search engine. For the labeled serum experiments, cysteine alkylation with the light form of acrylamide was set as a fixed modification and with the heavy form of acrylamide (+3.01884) as a variable modification. PeptideProphet [43] was used to evaluate each peptide assignment and ProteinProphet [44] was used to group peptides into protein groups, but only peptides having PeptideProphet probability >0.95 were used in the ProteinProphet inference (i.e., in contrast to the default configuration in ProteinProphet, we omitted moderately confident peptides due to the dramatic inflation of ProteinProphet rates that results from their inclusion [45]). Contaminant proteins were removed from the data set including those targeted for depletion, byproducts of coagulation or deleted from more recent IPI database releases. Relative quantitation was performed using the Q3 algorithm [29]. Peptides with zero area were reset to a background value to avoid singularities. Only highly confident peptide identifications – those having PeptideProphet probability ≥0.95, mass deviation <20 ppm and more than one scan – were used when computing protein ratios. Protein ratios were calculated by taking the geometric mean of all the associated peptide ratios for a protein group. Protein group ratios were logarithmically transformed and median-centered at zero. To quantify proteins from the peritoneal fluid experiments, total protein spectral count was used as a surrogate quantitative measure using only highly confident peptide identifications. In order to avoid singularities when computing ratios for the peritoneal fluid experiments, 0.5 as added to proteins with zero spectral counts in one fluid and nonzero counts in the other.

For each fluid, proteins from the two experiments were grouped using a previously described algorithm [46] so that each protein group contains one or more protein sequences indistinguishable based on the peptide evidence and consistent across experiments. Gene symbols were then assigned to each member of a protein group using IPI protein cross-reference and the protein groups were collapsed to a single gene symbol using the geometric mean for IPAS ratios and the maximum peptide counts for peritoneal fluid experiments. The serum and peritoneal fluid data sets were integrated by matching gene symbols.

### ELISA validation

Serum samples were screened for IGFBP1 and MMP9. IGFBP1 was evaluated using a DuoSet® ELISA development system from R&D Systems (Minneapolis, MN) according to the package directions. Briefly, the capture antibody was coated on an ELISA plate at 4 µg/ml in PBS overnight at room temperature. The wells were rinsed three times with wash buffer (0.05% Tween 20 in PBS) and blocked for 1 hour with blocking buffer (5% Tween 20 in PBS). The wells were washed again according to the previous directions. A standard curve was made in blocking buffer and serum samples were also diluted 1∶100 in blocking buffer. These samples were added to the plate in duplicate and allowed to incubate for 2 hours at room temperature. The wells were washed again and the detection antibody was added to the wells at 400 ng/ml. This was allowed to incubate for 2 hours and the wells were once again washed. Streptavidin-HRP was added and allowed to incubate for 20 minutes before the solution was removed and the wells washed. In order to develop the assay, TMB One Solution (Promega Corporation, Madison, WI) was added to the wells and allowed to incubate in the dark for 20 minutes before the reaction was stopped with an equal volume of 1N Sulfuric Acid (Acros, New Jersey). The optical density was determined using a microplate reader set to 450 nm. MMP9 was evaluated using the Human MMP-9 ELISA Kit from RayBiotech Inc. (Norcross, GA) according to the kit directions. Briefly, samples were diluted 1∶5000 and added along with standards to the plate precoated with the capture antibody. This was incubated for 2.5 hours at room temperature and then the wells were washed with Wash Buffer provided in the kit. The biotinylated detection antibody was added to the wells and incubated for 1 hour at room temperature. The wells were again washed with Wash Buffer and Streptavididn-HRP was added to the wells for 45 minutes. The wells were washed one final time and TMB One-Step Substrate Reagent was added to the wells and allowed to incubate for 30 minutes. The reaction was terminated by adding Stop Solution to the wells and the optical density was determined using a microplate reader set to 450 nm.

## Supporting Information

Table S1 Proteins up-regulated in either SOC peritoneal fluid or SOC serum.
(0.01 MB TXT)
